# The Needs of the Modern Health Resort

**Published:** 1904-06-04

**Authors:** 


### THE NEEDS OF THE MODERN HEALTH RESORT.

The value of any place as a health resort depends on either the possession of some natural water having therapeutic efficacy or on a climate having some special merit. When the former of these is the case it is still necessary to bear in mind the question of climate in dealing with the individual patient. For whilst as a spa the waters may be suitable for the disease, the climate may be unsuitable for the patient. In endeavouring to estimate climatic values meteorological observations no doubt have their place and value. But to the physician the knowledge of the effect of the climate on men and women is the first consideration, and a classification of climates from this point of view as bracing, sedative, or intermediate, is the main guide to practice. It is clinical or personal rather than meteorological or instrumental observations that are required. Granted a condition of climate possessing therapeutic properties it remains a necessity to supplement this by the resources of art. That public drainage and water supply should be above suspicion goes without saying. The same principles of supervision should be carried into the individual house, and both here and in such public reception rooms as concert halls, winter gardens, etc., efficient ventilation should be enforced by compulsory powers granted to the local public authority. The provision of popular attractions, though universally admitted in principle, is a problem difficult of solution in practice. It is necessary to remember the needs of the invalid and the convalescent, and at the same time not to forget that visitors who are taking a holiday need some appropriate amusements. The average British health station must be condemned as "appallingly and incurably dull." What is wanted is a *rendezvous* where all are more or less obliged to go at a given hour and where friends may be met and acquaintances made. This is best provided in the shape of a parade where social attractions can be cultivated in the open air and sunshine and under the enlivening influences of a good band. Protection from cold winds, and shelters on the esplanade and in the public parks and gardens should be provided, and these should be adequately inspected so as to secure their sanitary and æsthetic condition. Everything, in short, should be done to provide the visitor with the fullest opportunity to spend his time in the sunshine and fresh air. A further point needing attention is completeness of equipment for balneary and other allied forms of treatment. These are becoming of increasing importance, and the mere existence of excellent climatic conditions will not atone for their absence. Nauheim baths, massage, douches, radiant heat baths, and electricity in its various forms are all recognised adjuvants to climatic treatment in many forms of disease, and it is to the interest of all health stations which hope to command professional support and confidence to see that these can be obtained when desired. A properly organised institute is the only satisfactory method to secure this end, and such an equipment ought to be regarded as an essential need of a modern health resort. The stimulus to initiate the line of development above indicated must in many cases be provided by the local members of the profession, who alone are competent to estimate the needs of the situation. Similarly by mutual conference the medical men on the spot would do well to determine the *régime* to be followed by invalid visitors, and this more particularly in reference to diet. The influence of climate in this respect is imperfectly appreciated. But the exact effect of exercise, the necessary amount of sleep, and indeed many other details which together determine personal well-being are all influenced by local conditions, and it would be well if the practitioners in each locality could frame a joint conclusion on such points.

1 Leonard Williams: Journal of Balneology and Climatology, April, 1904.

